# Factors affecting prehospital time delay of the injured patients arriving at the Emergency Department of Beni-Suef University Hospital in Egypt: A cross-sectional study

**DOI:** 10.1371/journal.pone.0252044

**Published:** 2021-06-02

**Authors:** Doaa Mahmoud Khalil, Elmorsy Elmorsy, Ahmed Arafa, Hesham Ahmed Nafady, Lamiaa Saleh

**Affiliations:** 1 Department of Public Health and Community Medicine, Faculty of Medicine, Beni-Suef University, Beni-Suef, Egypt; 2 Public Health, Department of Social Medicine, Osaka University Graduate School of Medicine, Osaka, Japan; 3 Department of General Surgery, Faculty of Medicine, Beni-Suef University, Beni-Suef, Egypt; Sunnybrook Research Institute, CANADA

## Abstract

**Purpose:**

This study aimed to assess the factors affecting the prehospital time delay of the injured patients arriving at the Emergency Department of Beni-Suef University Hospital in Upper Egypt.

**Materials and methods:**

In this cross-sectional study, the following data were retrieved from the hospital records of 632 injured patients between 1/1/2018 and 31/3/2018: age, sex, residence, means of transportation to the hospital, prehospital time delay, consciousness level on admission, source of injury, and type of worst injury.

**Results:**

The prehospital time delay (>one hour) of the injured patients was positively associated with age >60 years and rural residence but inversely associated with consciousness level with odds ratios (95% confidence intervals) of 5.14 (2.26–11.68), 3.49 (2.22–5.48), and 0.56 (0.32–0.96), respectively.

**Conclusion:**

The prehospital time delay of the injured patients arriving at the Emergency Department of Beni-Suef University Hospital in Egypt was associated with old age, rural residence, and consciousness level.

## Introduction

Globally, the incidence of injuries increased during the period between 1990 and 2017 from 354 million to more than 520 million injuries. In 2017, injuries contributed to more than 4.5 million deaths, 3267 disability-adjusted life years (DALYs) lost per 100,000 people, more than 57 million years lived with disability (YLDs), and more than 195 million years of life lost (YLLs) [1]. Egypt carries the heaviest burden of injuries in the Middle East with high rates of injuries [2]. In 2017, the DALYs caused by injuries in Egypt reached 4000 years/100,000 people, and the YLDs and YLLs attributed to injuries represented 6% and 15% of all YLDs and YLLs in the country [1].

Several factors are related to the outcome of the injured patients such as the age of patients, the seriousness of the injury, the consciousness level, and prehospital time delay [3]. Based on these facts and given the wide difference in the injury outcome between low- and middle-income countries and high-income countries [4], determining the modifiable risk factors for unfavorable outcomes and in-depth investigation of their associations should be prioritized in low- and middle-income countries including Egypt. One of these factors; prehospital time delay and the resulting delay in the accessibility to emergency services can lead to delay in receiving proper management and consequently bad outcomes [5, 6].

A previous secondary data analysis study of prospective cohort trauma registry of patients attending a trauma center in California for 14 years, concluded that mortality increased in patients with penetrating injuries when the prehospital time was more than 20 minutes [7]. A survey study conducted on patients with isolated limb injuries in a trauma center in Montreal showed that delayed seeking orthopedic care largely affected the prognosis [8].

In Egypt, one study conducted at Ain Shams University Surgery Hospital in Cairo highlighted deficiency of trauma registry and inadequate communication between the ambulance system and the emergency departments. The study showed that among 149 poly-traumatized patients prehospital time delay was associated with knowing the local ambulance phone number, referral procedure, method of patients’ transfer, and type of injuries [9]. However, the health system in Upper Egypt is inferior to that in Cairo as there is a limited number of hospitals serving large numbers of populations with many structural, financial, and cultural barriers to health services [10]. Besides, there is still a lack of data about the factors related to the prehospital time delay in Upper Egypt. We, therefore, conducted this study to assess the factors affecting the prehospital time delay of the injured patients arriving at the Emergency Department of Beni-Suef University Hospital in Upper Egypt. Determining such associations may help in initiating new interventions to minimize prehospital time delay.

## Subjects and methods

### Study design and subjects

This cross-sectional analytical study was carried out at the Emergency Department of Beni-Suef University Hospital in Upper Egypt. It is one of the largest tertiary referral medical centers in South Egypt that belongs to the Ministry of Higher Education and provides free health services to about 3.2 million citizens [11].

The study subjects were all injured patients attending to the Emergency Department between 1/1/2018 and 31/3/2018 (the Emergency Department was working two days per week with average injured patients 25 per day, thus, the total number of the injured patients stood at 632, and all of them were included in this study. The sample size was estimated using the Epi- Info version 7 StatCalc designed by the Centers for Disease Control and Prevention (CDC) and the World Health Organization (WHO) based on the following expectations: prehospital time delay among injured patients admitted to the Emergency Department of Beni-Suef University Hospital at 25%, a margin of error of 5%, and population of 999,999. The least sample size was 289, however, we more than doubled the sample to avoid any unexpected decrease in the flow of patients, enhance the statistical power, and allow us to stratify the risk factors. After revising the records of the previous year, we could find that the required sample size can be collected in two to three months, therefore, we decided to collect records of the first three months of 2018.

### Data collection

The following data were retrieved from a predefined checklist for data collection from the Emergency Department records: age (years), sex (male or female), residence (urban or rural), consciousness on admission (conscious or semiconscious/unconscious), source of injury (road traffic crash, fall, shooting, burn, animal bite, or others), means of transportation to the hospital (ambulance or other means as private and public transport), type of worst injury (cut, laceration, contusion, burn, fracture, abdominal hemorrhage, cerebral hemorrhage, or others), and prehospital time delay (minutes). Data were collected by the first author and the head nurse of the Emergency Department.

The consciousness level was primarily assessed using the Alert, Verbal, Pain, and Unresponsive (AVPU) score; a system used by a health care professional to measure and record a patient’s level of consciousness, and it is mostly used in emergency protocols and first aid scenes. Patients with A and V were considered conscious and patients with VU were considered semiconscious/unconscious [12]. The prehospital time delay, which was considered as the outcome in this study, was defined as the time from the occurrence of the injury until reaching the Emergency Department of Beni-Suef University Hospital.

### Statistical analysis

The prehospital time was categorized according to its median value into ≤ or > one hour where >one hour was defined as the prehospital time delay. It has long been concluded that the first hour after the injury is called the “golden hour” because early proper management can save injured patients from severe morbidity and death [13]. Binary logistic regression analysis was used to compute odds ratios (ORs) and their corresponding 95% confidence intervals (95% CIs) for the associations between (age, sex, residence, source of injury, type of worst injury, and consciousness level) and prehospital time delay. Data were analyzed using SPSS version 25.

### Ethical considerations

The study protocol was reviewed by the Beni-Suef University Research Ethics Committee and given approval number: FMBSRUEC/07062020/Khalil. The study was conducted per the principles of the Declaration of Helsinki.

## Results

Out of the 632 injured patients, 30.4% were aged <15 years, 67.4% were males, 70.7% were residing in rural areas. Almost a third of patients (34.8%) reported transportation to the hospital via ambulance. Most patients (69.3%) reached the hospital within one hour of injury and 30.7% reached after one hour of injury representing the prevalence of prehospital time delay in this study. Road traffic crashes represented half of the injury sources (Table 1).

**Table 1:** Sociodemographic and clinical characteristics of the injured patients.

Characteristics		Overall n = 632 (%)
Age (years)	< 15	192 (30.4)
	15 to ≤40	337 (53.3)
	>40 to ≤60	75 (11.9)
	> 60	28 (4.4)
Sex	Male	426 (67.4)
	Female	206 (32.6)
Residence	Rural	447 (70.7)
	Urban	185 (29.3)
Mean of transportation to the hospital	Ambulance	220 (34.8)
	Others#	412 (65.2)
Hospital arrival time	≤ One hour	438 (69.3)
	> One hour	194 (30.7)
Source of injury	Road traffic crash	317 (50.2)
	Fall	202 (32.0)
	Shooting	10 (1.6)
	Burn	10 (1.6)
	Animal bite	17 (2.6)
	Others*	76 (12.0)
Type of worst injury	Cut/laceration/contusion	302 (47.8)
	Fracture	186 (29.4)
	Burn	10 (1.6)
	Abdominal hemorrhage	27 (4.3)
	Cerebral hemorrhage	29 (4.6)
	Others¶	78 (12.3)
Consciousness level	Conscious	546 (86.4)
	Semiconscious/unconscious	86 (13.6)

^#^Others in the mean of transportation refer to private cars, general transportation, and walking

*Others in the source of injury refer to struggle, injury with sharp objects, occupational injuries, and falling of an object

^¶^Others in the type of worst injury refer to fracture base of the skull, vertebral column injury, and amputation of a limb.

Older patients (>60 years) were more susceptible to prehospital time delay compared with middle-aged patients (OR: 5.14, 95% CI 2.26–11.68). Patients residing in rural areas reported a higher likelihood of prehospital time delay in comparison with those with urban residence (OR: 3.49, 95% CI 2.22–5.48). Patients diagnosed as semiconscious/unconscious on admission were less likely to be subjected to prehospital time delay compared with conscious patients (OR: 0.56, 95% CI 0.32–0.96) (Table 2).

**Table 2:** Associations with the prehospital delay of the injured patients.

Characteristics		≤ One hour	> One hour	OR (95% CI)
Age	< 15	137 (71.4)	55 (28.6)	0.97 (0.67–1.43)
	15 to ≤60	292 (70.9)	120 (29.1)	1
	> 60	9 (32.1)	19 (67.9)	**5.14 (2.26**–**11.68)**
Sex	Male	302 (70.9)	124 (29.1)	0.79 (0.56–1.14)
	Female	136 (66.0)	70 (34.0)	1
Residence	Rural	280 (62.6)	167 (37.4)	**3.49 (2.22–5.48)**
	Urban	158 (85.4)	27 (14.6)	1
Mean of transportation to the hospital	Others#	280 (68.0)	132 (32.0)	1.20 (0.84–1.72)
	Ambulance	158 (71.8)	62 (28.2)	1
Source of injury	Road traffic crash	218 (68.8)	99 (31.2)	0.86 (0.55–1.36)
	Fall	146 (72.3)	56 (27.7)	0.73 (0.44–1.19)
	Others*	74 (65.5)	39 (34.5)	1
Type of worst injury	Cut/laceration/contusion	226 (74.8)	76 (25.2)	0.67 (0.44–1.04)
	Fracture	116 (62.4)	70 (37.6)	1.20 (0.77–1.90)
	Others¶	96 (66.7)	48 (33.3)	1
Consciousness level	Semiconscious/unconscious	68 (79.1)	18 (20.9)	**0.56 (0.32**–**0.96)**
	Conscious	370 (67.8)	176 (32.2)	1

OR: Odds ratio

CI: Confidence interval

^#^Others in the mean of transportation refer to private cars, general transportation, and walking

*Others in the source of injury refer to struggle, injury with sharp objects, occupational injuries, and falling of an object

^¶^Others in the type of worst injury refer to fracture base of the skull, vertebral column injury, and amputation of a limb.

## Discussion

Emergency service is an integrated system to deliver time-sensitive health services for acute illness and injury. The emergency care system that delivers these services extends from care at the site of injury throughout transport to the emergency department. Many health interventions are highly dependent on time to save lives, but only when delivered at the proper time [14]. This study indicated some associations with prehospital time delay among the injured patients arriving at the Emergency Department of Beni-Suef University Hospital in Upper Egypt. Age >60 years and residing in rural areas were associated with prehospital time delay. Semiconsciousness/unconsciousness patients were more likely to arrive earlier than conscious patients.

The results indicated that the old age of injured patients in Beni-Suef was associated with prehospital time delay. In line with the current findings, a previous study showed that older patients with stroke seeking emergency care in Taiwan were more susceptible to prehospital time delay [15]. Another study showed that older patients who presented with ST-elevation myocardial infarction arrived later at the Emergency Department in comparison with their younger counterparts with the same condition [16]. The association between prehospital time delay and old age is unclear but it could be suggested that many older patients live alone and suffer denial and economic problems; factors that may postpone their arrival to the hospital [17].

Moreover, the rural residence of the studied patients was associated with a high likelihood of prehospital time delay which agreed with a previous study from India. This association could be explained by the increased illiteracy rates in rural areas, lack of access to transportation, and distant nearby medical services [16]. In Egypt, the rural-urban discrepancies in access to healthcare are one of the striking defects of the Egyptian healthcare system that may partly explain the prehospital time delay among patients residing in rural areas [18]. Also, there was a lack of optimal development in rural areas and a lack of affordability of different forms of healthcare including emergency health services [19]. Also, the higher illiteracy rate in rural areas compared with urban areas in Beni-Suef [11] might have attributed to this association.

Surprisingly, the disturbed consciousness level of the injured patients contributed to less susceptibility to prehospital time delay. This finding agreed with previous studies conducted on patients with brain trauma [20, 21]. The conscious patients could be more susceptible to prehospital time delay due to the absence of external features that can be a source of worry for a hurry to seek emergency care [20, 21].

Although this study assessed the associations with prehospital time delay among a large study population, a few limitations should be addressed. First, like most hospitals in Egypt, Beni-Suef University Hospital suffers a lack of health records including reliable personal and clinical data of patients and events from the occurrence of injury till reaching the hospital. For example, no hospital records included data on the vital signs at the scene of the injury and first aids done. Therefore, the authors depended on the data recorded by the staff of the Emergency Department after patient arrival. Second, due to logistic problems, the Emergency Department during the study period was working for two days per week, therefore, the authors couldn’t assess the associations with prehospital time delay among all injured patients throughout the study period as they received emergency care in other hospitals.

## Conclusion

This study concluded that prehospital time delay was associated with old age, rural residence, and consciousness level. Thus, conducting health education programs targeting old people and people residing in rural areas about the seriousness of prehospital delay is warranted.

## Supporting information

S1 File (XLSX)

## References

[1] James SL, Lucchesi LR, Bisignano C, Castle CD, Dingels ZV, Fox JT, et al., Morbidity and mortality from road injuries: results from the Global Burden of Disease Study 2017. Injury prevention. 2020 1 8. 10.1136/injuryprev-2019-043302 PMC7571357 31915274

[2] Arafa A, El-Setouhy M, Hirshon JM. Driving behavior and road traffic crashes among professional and nonprofessional drivers in South Egypt. International journal of injury control and safety promotion. 2019 10 2;26(4):372–8. 10.1080/17457300.2019.1638419 31282807 PMC6851424

[3] Yu W, Chen H, Lv Y, Deng Q, Kang P, Zhang L. Comparison of influencing factors on outcomes of single and multiple road traffic injuries: A regional study in Shanghai, China (2011–2014). PloS one. 2017 5 11;12(5):e0176907. 10.1371/journal.pone.0176907 28493893 PMC5426634

[4] WHO (World Health Organization). Violence and injury prevention, WHO trauma care and services publications and resource. 2014. https://www.who.int/violence_injury_prevention/publications/services/en/. Accessed on 10/7/2020.

[5] Harrington DT, Connolly M, Biffl WL, Majercik SD, Cioffi WG. Transfer times to definitive care facilities are too long: a consequence of an immature trauma system. Annals of surgery. 2005 6;241(6):961. 10.1097/01.sla.0000164178.62726.f1 15912045 PMC1357175

[6] Sakran JV, Greer SE, Werlin E, McCunn M. Care of the injured worldwide: trauma still the neglected disease of modern society. Scandinavian journal of trauma, resuscitation and emergency medicine. 2012 12;20(1):1–6. 10.1186/1757-7241-20-64 22980446 PMC3518175

[7] McCoy CE, Menchine M, Sampson S, Anderson C, Kahn C. Emergency medical services out-of-hospital scene and transport times and their association with mortality in trauma patients presenting to an urban Level I trauma center. Annals of emergency medicine. 2013 2 1;61(2):167–74. 10.1016/j.annemergmed.2012.08.026 23142007

[8] Rouleau DM, Feldman DE, Parent S. Delay to orthopedic consultation for isolated limb injury: cross-sectional survey in a level 1 trauma centre. Canadian Family Physician. 2009 10 1;55(10):1006–7. 19826162 PMC2761960

[9] Saleh HM, Elsabagh AE, Elewa MG, Fawzy AA, Hassan OM, Comer AC, et al., Admission delays’ magnitude of traumatized patients in the emergency department of a hospital in Egypt: a cross-sectional study. European journal of trauma and emergency surgery. 2018 4 1;44(2):225–30. 10.1007/s00068-017-0762-1 28255612 PMC5582022

[10] Chiang C, Labeeb SA, Higuchi M, Mohamed AG, Aoyama A. Barriers to the use of basic health services among women in rural southern Egypt (Upper Egypt). Nagoya journal of medical science. 2013 8;75(3–4):225. 24640178 PMC4345669

[11] CAPMAS. Central Agency for Public Mobilization and Statistics. Egypt statistics. Final results of 2017 Census.2018. http://www.capmas.gov.eg. Accessed on 5/7/2020.

[12] McNarry AF, Goldhill DR. Simple bedside assessment of level of consciousness: comparison of two simple assessment scales with the Glasgow Coma scale. Anaesthesia. 2004 1;59(1):34–7. 10.1111/j.1365-2044.2004.03526.x 14687096

[13] Newgard CD, Meier EN, Bulger EM, Buick J, Sheehan K, Lin S, et al., ROC Investigators. Revisiting the “golden hour”: an evaluation of out-of-hospital time in shock and traumatic brain injury. Annals of emergency medicine. 2015 7 1;66(1):30–41. 10.1016/j.annemergmed.2014.12.004 25596960 PMC4478150

[14] WHO (World Health Organization). Global Health Estimates 2016: Disease burden by cause, age, sex, by country and by region, 2000–2016, Geneva. 2018. https://www.who.int/healthinfo/global_burden_disease/estimates/en/index1.html. Accessed on 15/7/2020.

[15] Chang KC, Tseng MC, Tan TY. Prehospital delay after acute stroke in Kaohsiung, Taiwan. Stroke. 2004 3 1;35(3):700–4. 10.1161/01.STR.0000117236.90827.17 14963279

[16] Mohan B, Bansal R, Dogra N, Sharma S, Chopra A, Varma S, et al. Factors influencing prehospital delay in patients presenting with ST-elevation myocardial infarction and the impact of prehospital electrocardiogram. Indian Heart Journal. 2018 12 1;70:S194–8. 10.1016/j.ihj.2018.10.395 30595256 PMC6309871

[17] Nguyen H. L., Saczynski J. S., Gore J. M., & Goldberg R. J. (2010). Age and sex differences in duration of prehospital delay in patients with acute myocardial infarction: a systematic review. Circulation: Cardiovascular Quality and Outcomes, 3(1), 82–92. 10.1161/CIRCOUTCOMES.109.884361 20123674 PMC3072277

[18] Boutayeb A, Helmert U. Social inequalities, regional disparities and health inequity in North African countries. International Journal for Equity in Health. 2011 12 1;10(1):23. 10.1186/1475-9276-10-23 21627818 PMC3120653

[19] Elgazzar H. Income and the use of health care: an empirical study of Egypt and Lebanon. Health Econ. Pol’y & L. 2009;4:445. 10.1017/S1744133109004939 19254431

[20] MacKenzie EJ, Rivara FP, Jurkovich GJ, Nathens AB, Frey KP, Egleston BL, et al., A national evaluation of the effect of trauma-center care on mortality. New England Journal of Medicine. 2006 1 26;354(4):366–78. 10.1056/NEJMsa052049 16436768

[21] Raj R, Siironen J, Kivisaari R, Kuisma M, Brinck T, Lappalainen J, etal., Factors correlating with delayed trauma center admission following traumatic brain injury. Scandinavian journal of trauma, resuscitation and emergency medicine. 2013 12 1;21(1):67. 10.1186/1757-7241-21-67 24020630 PMC3846883

